# Exploring the Link Between Adjacent Lateral Incisor Morphology and Unilateral Maxillary Canine Impaction: A Cone-Beam Computed Tomography Study

**DOI:** 10.7759/cureus.76806

**Published:** 2025-01-02

**Authors:** Susmita Majumder, Shiraz Siddiqui, Mohsin A Wani, Sakina Sattar, Firdosh Rozy, Sunegha Kundal, Seema Gupta

**Affiliations:** 1 Department of Orthodontics, Aditya Diagnostics and Hospital, Dibrugarh, IND; 2 Department of Orthodontics, Health Gulf Medical Company Limited, Tabuk, SAU; 3 Department of Orthodontics, Apple Bite Clinics, Allahabad, IND; 4 Department of Orthodontics, Rural Dental College, Pravara Institute of Medical Sciences (Deemed to be University), Ahmednagar, IND; 5 Department of Orthodontics, Dental Care Complex, Riyadh, SAU; 6 Department of Orthodontics, Kanti Devi Dental College and Hospital, Mathura, IND; 7 Department of Orthodontics, Kothiwal Dental College and Research Centre, Moradabad, IND

**Keywords:** cone-beam computed tomography, impacted maxillary canine, maxillary lateral incisor, morphology, unilateral

## Abstract

Introduction: The maxillary canine plays a pivotal role in dental aesthetics, occlusion, and function because of its strategic anatomical positioning. However, the eruption of maxillary canines is a complex process that often leads to impaction. The anatomical and morphological characteristics of adjacent teeth, particularly the maxillary lateral incisors (MLIs), are believed to influence the eruption of maxillary canines. This study aimed to investigate the association between the morphology of MLI and unilateral impacted maxillary canines (IMCs) while also identifying potential predictors of such impactions.

Materials and methods: This retrospective study analyzed cone-beam computed tomography (CBCT) scans of 40 patients with unilateral IMC conducted between January 2020 and December 2023. Patients were selected based on the inclusion criteria that required intact MLIs adjacent to both impacted and non-impacted canines. CBCT imaging was performed using standardized protocols to assess crown dimensions, root length, angulation, and spatial relationships of the lateral incisors. The measurements were recorded independently by two calibrated observers. Statistical analyses included independent t-tests and multivariate logistic regression, with significance set at p < 0.05.

Results: Significant differences were observed in the morphological parameters of the MLI between the impacted and non-impacted sides. Root length and full length were significantly smaller on the impacted side (p < 0.05), whereas mesiodistal (MD) dimensions showed no significant difference. Labiolingual (LL) dimensions and angular measurements revealed significant variations, with mesially inclined and labially displaced roots on the impacted side. Sex differences were noted, with females exhibiting smaller root lengths and buccolingual (BL) dimensions than males. Multivariate logistic regression identified sex, root length, MD width, and angulation with the mid-sagittal plane (MSP) as significant predictors of IMC.

Conclusion: Unilateral IMC were significantly associated with smaller root lengths, LL dimensions, and mesially inclined roots of MLI.

## Introduction

The maxillary canine (MC) is instrumental in aesthetics, functionality, and occlusal harmony owing to its strategic anatomical positioning, elongated root structure, and salient location within the smile line, rendering it an essential element of the dental arch. Nevertheless, the processes governing its development and eruption trajectory are intricate and frequently accompanied by considerable challenges. Impacted maxillary canines (IMCs) occur at a rate of 1.38%, which means that roughly one in 72 people may experience this condition. This prevalence varies slightly across populations and may be influenced by genetic, environmental, and developmental factors [1]. Moreover, empirical observations demonstrate that the frequency of IMC is three to six times greater on the palatal side than on the buccal side [2]. However, divergent research findings suggest that within East Asian demographics, IMC is reported to occur two to three times more commonly on the buccal aspect as opposed to the palatal aspect [3]. The etiology of IMC has garnered substantial scholarly attention, with investigations highlighting the roles of genetic, anatomical, and environmental determinants.

Among these factors, the morphological characteristics of the adjacent dentition, particularly the maxillary lateral incisor (MLI), have received considerable attention. The anatomical features of both the crown and root of MLI are posited to exert influence on the eruption trajectory of the neighboring MC. Variations in the dimensions, morphology, and angulation of the MLI can modify spatial and developmental interactions, thereby potentially contributing to IMC [4]. For instance, peg-shaped MLIs or those exhibiting diminished dimensions have been correlated with an elevated incidence of IMC. Likewise, root morphology and positioning of the MLI may establish physical impediments or facilitate the eruption course of the MC, thereby further complicating its developmental trajectory [5,6].

The association between the morphology of MLIs and IMC has considerable significance in both diagnostic and therapeutic approaches [7,8]. Gaining insight into this relationship can facilitate the early identification of potential impacts, thereby enabling timely interventions to direct the eruption trajectory of the MC. Furthermore, it contributes to the strategic planning of orthodontic and restorative interventions, thereby ensuring the holistic management of the dental arch. Notwithstanding its critical relevance, this association continues to be the subject of ongoing investigation, with numerous facets yet to be completely clarified.

Unilateral IMC provides a distinctive framework for investigating this correlation. In contrast to bilateral impaction, where systemic or genetic influences may prevail, unilateral impaction facilitates a more precise analysis of site-specific factors, including the anatomical characteristics of the neighboring MLI [9]. By examining the crown and root structures of MLIs adjacent to both impacted and non-impacted MC within the same subject, researchers can derive a significant understanding of the etiological implications of MLI morphology in the context of IMC.

This study aimed to explore the relationship between the morphological features of the crowns and roots of MLIs and the incidence of unilateral IMC through the utilization of cone-beam computed tomography (CBCT). A secondary objective was to identify predictors of unilateral IMC.

## Materials and methods

Study design and setting

This retrospective study used CBCT records collected from patients who underwent imaging at the Kothiwal Dental College and Research Centre, Moradabad, between January 2020 and December 2023. The study was approved by the Institutional Ethical Review Board (approval number: KDCRC/IERB/02/2024/69), and the requirement for written informed consent was waived owing to the retrospective design. All patient data were anonymized to maintain confidentiality, in accordance with the Declaration of Helsinki.

Sample size estimation

The sample size estimation for this study was calculated using G*Power software (version 3.6.9; Heinrich Heine University Düsseldorf, Düsseldorf, Germany) with a power of 80% and an alpha error of 5%. The effect size of 0.56 was derived from a reference study, which examined the mean difference in the angle of the MLI from the occlusal plane between the impacted and non-impacted sides of the MC [10]. Based on these parameters, the estimated sample size was 80, which corresponds to CBCT records from 40 patients, as the study design adopted a split-mouth approach.

Patients’ eligibility criteria

The study population included patients aged 12-40 years who had CBCT scans of sufficient quality to visualize the MCs and MLIs in detail. Inclusion criteria required the presence of unilateral IMC and intact MLIs adjacent to both impacted and non-impacted MCs. Exclusion criteria were syndromic conditions, cleft lip or palate, or any missing or previously extracted MLIs. Additionally, patients with prior orthodontic or surgical interventions affecting the MCs were excluded, as were those with CBCT scans with artifacts or inadequate resolution.

Methodology

CBCT imaging was performed using the Carestream new generation CBCT apparatus (Carestream Dental, Atlanta, GA) in accordance with a standardized protocol. Scans were conducted with patients in a natural head position to allow accurate representation of anatomical structures. Imaging parameters included a field of view appropriate for capturing the maxillary dentition, a voxel size of 0.2 mm, a tube voltage of 90 kVp, a tube current of 8 mA, a slice thickness of 0.2 mm, and a field of view (FOV) measuring 8 x 8 cm.

**Figure 1:**
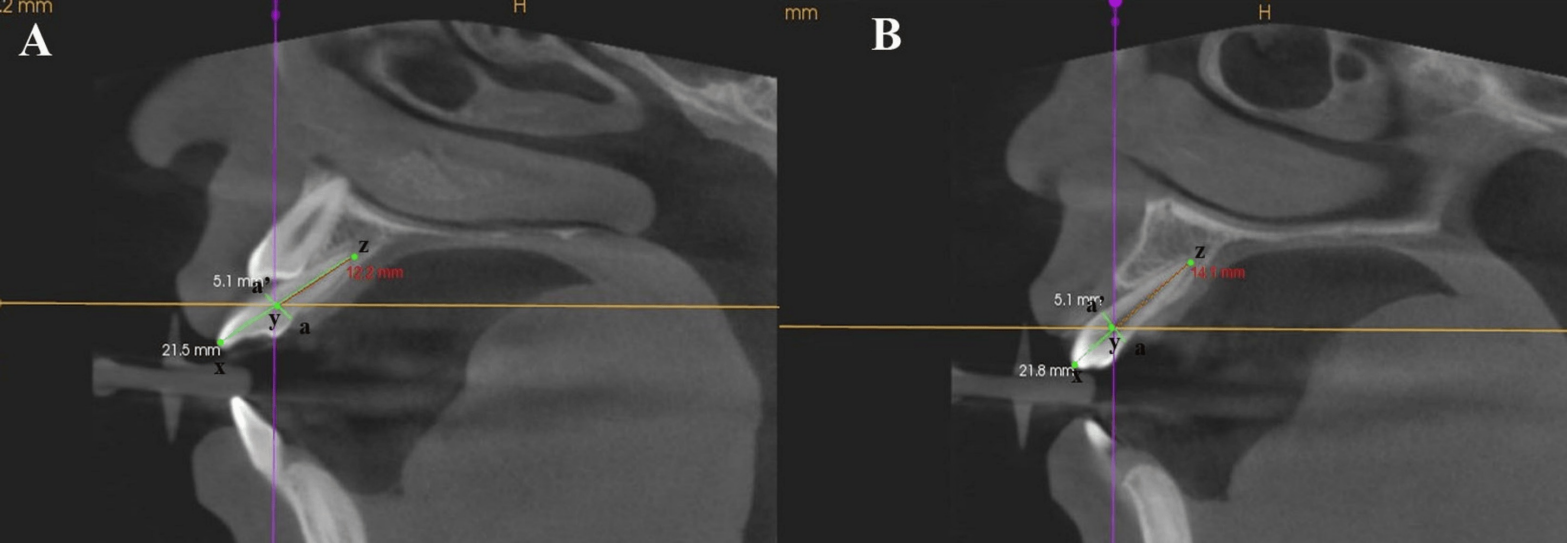
Incisoapical length of the maxillary lateral incisor (xz), root length (yz), and labiolingual width of the crown (aa') in sagittal view adjacent to the impacted maxillary canine (A) and adjacent to non-impacted maxillary canine (B).

**Figure 2:**
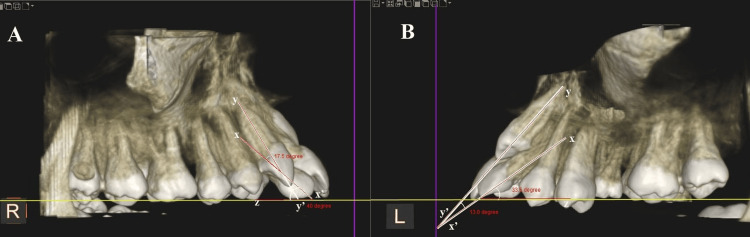
Angle (xx'z) between the maxillary long axis of the maxillary lateral incisor (xx') and occlusal plane (z line), and angle between the long axis of maxillary canine (yy') and maxillary lateral incisor (xx') on non-impacted side (A) and impacted side (B) in sagittal view.

**Figure 3:**
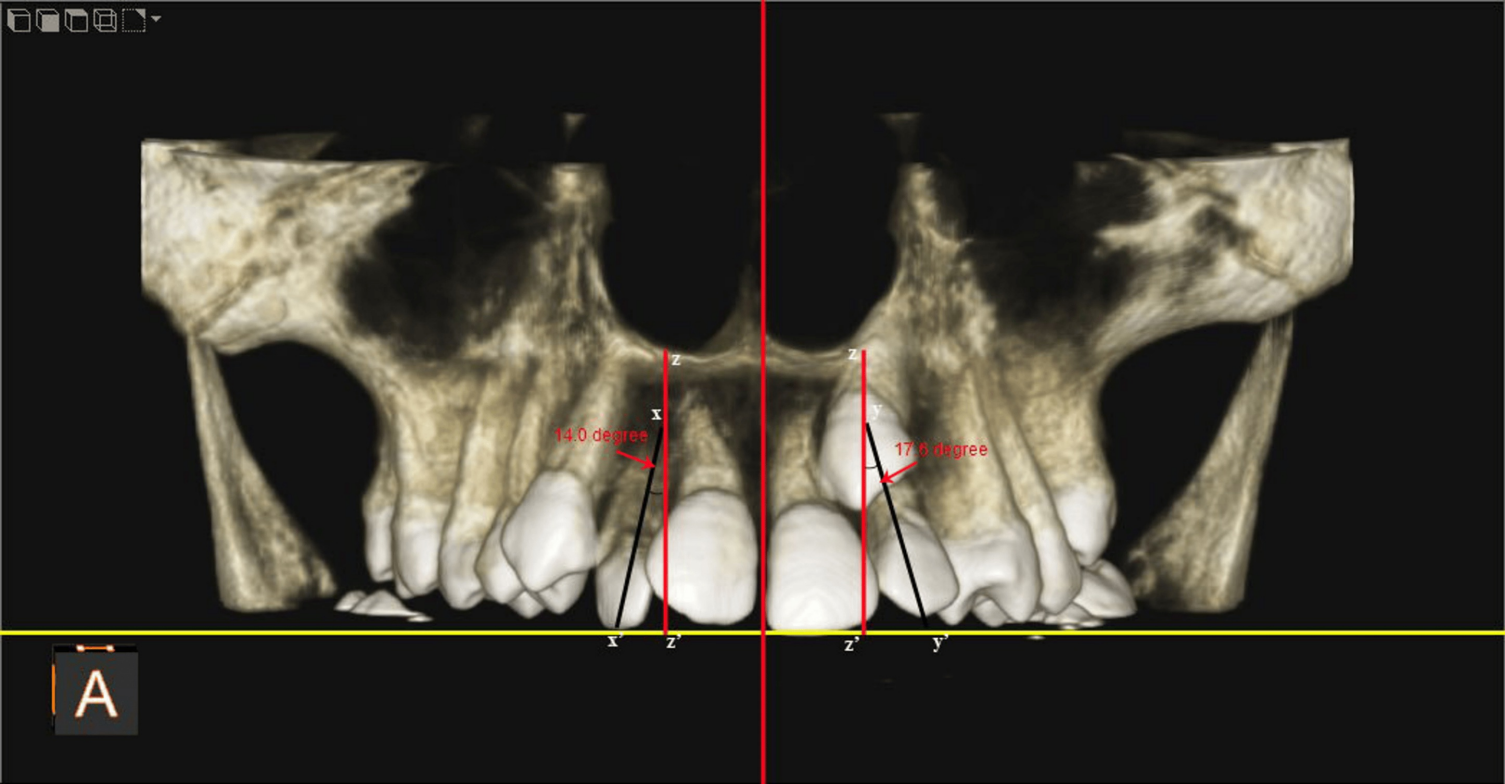
Angulation of the long axis of the maxillary lateral incisor (xx') on the non-impacted side and impacted side (yy') with a mid-sagittal plane (xz') in coronal view.

Morphological assessments were conducted using Carestream software, which facilitates three-dimensional visualization and precise measurements. The analysis focused on the MLIs adjacent to both impacted and non-impacted MCs. The parameters of interest included crown dimensions (mesiodistal (MD) width and labiolingual (LL) thickness) and complete tooth length (incisoapical length). The root morphology of MLI was evaluated for length (Figure 1). The angulation of MLI relative to the occlusal plane (OP), with a long axis of canine (Figure 2), and with respect to the mid-sagittal plane (MSP) (Figure 3) was also evaluated.

MLIs were classified into two groups: those adjacent to the IMC (impacted group) and those adjacent to non-impacted MCs (control group). The measurements were performed independently by two calibrated observers. Both observers were told about the detailed method of measurements, and 20 CBCT scans were provided for reliability testing. The intraclass correlation coefficients (ICCs) revealed high reliability. After calibration, both observers evaluated all CBCT scans independently. To ensure measurement accuracy and reliability, all parameters were re-evaluated by the same observers after a two-week interval, and intra- and inter-observer reliabilities were assessed. This methodological approach enabled a detailed evaluation of the morphological characteristics of MLIs and their potential association with the occurrence of unilateral IMC. Reliability was evaluated by measuring intra- and inter-observer agreement using ICCs, with ICC values greater than 0.80 indicating high reliability.

Statistical analysis

Descriptive statistics, including mean and standard deviation, was used to summarize continuous variables. Data distribution was assessed for normality using the Shapiro-Wilk test, and the data were found to be normally distributed. Statistical comparisons between the impacted and non-impacted groups were conducted using independent t-tests for continuous variables. A linear regression analysis was performed for outcome variables on the impacted and non-impacted sides to analyze the effects, with a significance threshold of p < 0.05. Statistical analyses were performed using SPSS version 23.0 software (IBM Corp., Armonk, NY).

## Results

A descriptive analysis of the variables for the impacted and non-impacted sides was performed for the male and female groups. The root length of MLI was greater in males than in females, and it was smaller on the impacted side in both sexes than on the non-impacted side. The full length of the MLI was similar between sexes, with greater length on the non-impacted side. In terms of the LL thickness of the crown of the MLI, males exhibited a greater LL thickness on the impacted side, whereas females exhibited greater buccolingual (BL) thickness on the non-impacted side. The MD width of the crown of the MLI was consistent between the sexes, with a smaller MD width on the impacted side in both sexes. For angular measurements, the angle of the long axis of the MLI with the OP was smaller on the impacted side, showing labial inclination of the MLI, than on the non-impacted side in both sexes. The angle of the long axis of the MLI with the MSP was greater on the impacted side, having mesially inclined roots, compared to the non-impacted side, and this angle was greater in females on the impacted side. Lastly, the angle between the long axis of the MLI and the MC showed a smaller value on the impacted side, showing the closeness of the crown of the MC with the root of the MLI.

These results highlight distinct variations between the impacted and non-impacted sides across both sexes, with notable differences in root length, angle, and width. The sex differences were statistically significant for all the variables for both groups, except for the MD width of the MLI (p < 0.05), as shown in Tables 1, 2.

**Table 1:** Descriptive analysis of variables for sexes between impacted and non-impacted sides. Data are represented in the form of mean and standard deviation (SD). LI: lateral incisor; LL: labiolingual; MD: mesiodistal; OP: occlusal plane; MSP: mid-sagittal plane; CI: confidence interval.

Descriptive statistics			Impacted side				Non-impacted side			
Variables	Sex	N	Mean	SD	95% CI mean		Mean	SD	95% CI mean	
					Upper	Lower			Upper	Lower
Age in years	Male	20	20.25	2.73	21.52	18.97	20.25	2.73	21.52	18.97
	Female	20	19.70	3.19	21.19	18.20	19.70	3.19	21.19	18.20
Root length of LI in mm	Male	20	11.69	0.97	12.15	11.23	13.16	0.67	13.48	12.85
	Female	20	11.65	1.16	12.19	11.10	13.08	0.77	13.44	12.72
Full length of LI in mm	Male	20	21.03	1.04	21.52	20.54	22.28	0.77	22.64	21.92
	Female	20	21.06	1.24	21.64	20.47	22.28	0.91	22.70	21.85
LL thickness of LI crown in mm	Male	20	5.10	0.33	5.26	4.94	5.31	0.26	5.43	5.18
	Female	20	5.05	0.37	5.22	4.88	5.35	0.33	5.51	5.19
MD width of LI crown in mm	Male	20	6.08	0.12	6.13	6.02	6.11	0.11	6.16	6.05
	Female	20	6.09	0.11	6.15	6.04	6.13	0.09	6.18	6.09
Angle of LI with OP in LL direction (°)	Male	20	34.37	2.45	35.52	33.22	42.53	1.36	43.17	41.89
	Female	20	34.61	2.48	35.77	33.45	42.48	1.54	43.20	41.75
Angle of LI with MSP in MD direction (°)	Male	20	18.14	1.02	18.61	17.66	14.69	0.88	15.11	14.28
	Female	20	18.46	0.92	18.89	18.02	14.47	0.86	14.87	14.07
Angle of LI with canine (°)	Male	20	13.21	0.61	13.5	12.92	17.62	0.93	18.05	17.18
	Female	20	13.07	0.62	13.36	12.77	17.41	0.93	17.85	16.97

**Table 2:** Comparison outcome variables for sexes between impacted and non-impacted sides. * P-value < 0.05: significant. LI: lateral incisor; LL: labiolingual; MD: mesiodistal; OP: occlusal plane; MSP: mid-sagittal plane.

Variables	Impacted side		Non-impacted side	
	t-value	P-value using an independent t-test	t-value	P-value using an independent t-test
Root length of LI in mm	-5.546	0.001*	-4.585	0.001*
Full length of LI in mm	-4.288	0.001*	-3.538	0.001*
LL thickness of LI crown in mm	-2.157	0.037*	-2.665	0.011*
MD width of LI crown in mm	-0.836	0.408	-1.267	0.213
Angle of LI with OP in LL direction (°)	-13.004	0.001*	-12.032	0.001*
Angle of LI with MSP in MD direction (°)	11.375	0.001*	14.06	0.001*
Angle of LI with canine (°)	-17.559	0.001*	-17.301	0.001*

The comparison of the impacted and non-impacted groups using independent t-tests revealed significant differences for most variables. The mean root length of the MLI was significantly smaller for the impacted side (11.67 ± 1.06 mm) than for the non-impacted side (13.12 ± 0.72 mm). Similarly, the mean full length of the MLI was significantly smaller on the impacted side (21.05 ± 1.13 mm) than on the non-impacted side (22.28 ± 0.84 mm). For BL width, the impacted side measured significantly less (5.08 ± 0.35 mm), while the non-impacted side measured 5.33 ± 0.30 mm. MD width showed no significant difference between the groups (p > 0.05). Angular measurements displayed significant disparities. The angle with the OP was lower on the impacted side than on the non-impacted side (p = 0.001). The angle with the MSP was significantly lower on the impacted side (18.3 ± 0.98°) than on the non-impacted side (14.59 ± 0.87°). Finally, the angle with the MC was 13.14 ± 0.62° for the impacted side and 17.52 ± 0.93° for the non-impacted side (p = 0.001). These results highlight the significant morphological differences between the impacted and non-impacted sides across most parameters (Table 3).

**Table 3:** Comparison outcome variables between impacted and non-impacted sides. * P-value < 0.05: significant. Data are represented in the form of mean ± standard deviation (SD). LI: lateral incisor; LL: labiolingual; MD: mesiodistal; OP: occlusal plane; MSP: mid-sagittal plane; CI: confidence interval; L: lower limit; U: upper limit.

Variables	Group	Minimum	Maximum	95% CI for mean (L-U)	Mean ± SD	t-value	P-value using an independent t-test
Root length of LI in mm	Impacted	10.10	13.30	11.33 - 12.01	11.67 ± 1.06	7.17	0.001*
	Non-impacted	11.50	14.10	12.89 - 13.35	13.12 ± 0.72		
Full length of LI in mm	Impacted	19.30	22.70	20.68 - 21.41	21.05 ± 1.13	5.54	0.001*
	Non-impacted	20.60	23.40	22.01 - 22.55	22.28 ± 0.84		
LL thickness of LI crown in mm	Impacted	4.56	5.67	4.97 - 5.19	5.08 ± 0.35	3.46	0.001*
	Non-impacted	4.80	5.80	5.24 - 5.43	5.33 ± 0.3		
MD width of LI crown in mm	Impacted	5.89	6.31	6.05 - 6.13	6.09 ± 0.11	1.48	0.145
	Non-impacted	5.89	6.34	6.09 - 6.16	6.12 ± 0.1		
Angle of LI with OP in LL direction (°)	Impacted	30.50	39.20	33.71 - 35.28	34.49 ± 2.44	17.9	0.001*
	Non-impacted	40.01	44.70	42.05 - 42.97	42.51 ± 1.44		
Angle of LI with MSP in MD direction (°)	Impacted	16.40	19.6	17.99 - 18.61	18.3 ± 0.98	17.94	0.001*
	Non-impacted	13.40	15.90	14.31 - 14.86	14.59 ± 0.87		
Angle of LI with canine (°)	Impacted	12.40	14.30	12.94 - 13.34	13.14 ± 0.62	24.81	0.001*
	Non-impacted	16.20	18.90	17.22 - 17.81	17.52 ± 0.93		

Linear regression analysis identified the significant predictors of the variables examined. Sex was a significant factor, with males (p = 0.005) and females (p = 0.006) demonstrating significant associations with the outcome. Root length showed a negative relationship (p = 0.041), indicating that shorter root length was a significant predictor. Conversely, full-length (p = 0.029) and MD width (p = 0.034) were positively associated, suggesting their contributions to the dependent variable. The angle with the MSP was negatively correlated (p = 0.018), whereas the angle with the MC showed the strongest positive association (p < 0.001), highlighting its importance in the model. No significant associations were observed between LL thickness (p = 0.194) or angle and OP (p = 0.155), as shown in Table 4.

**Table 4:** Linear regression analysis for outcome variable. * P-value < 0.05: significant. LI: lateral incisor; LL: labiolingual; MD: mesiodistal; OP: occlusal plane; MSP: mid-sagittal plane.

Variables	Unstandardized coefficient	t-value	P-value
Sex (M)	-5.451	-2.907	0.005*
Sex (F)	-5.379	-2.858	0.006*
Root length of LI in mm	-0.168	-2.088	0.041*
Full length of LI in mm	0.157	2.228	0.029*
LL thickness of LI crown in mm	0.093	1.311	0.194
MD width of LI crown in mm	0.541	2.163	0.034*
Angle of LI with OP in LL direction (°)	0.016	1.437	0.155
Angle of LI with MSP in MD direction (°)	-0.054	-2.429	0.018*
Angle of LI with canine (°)	0.133	6.393	0.001*

## Discussion

The present study was conducted to assess the morphological parameters of MLI as predictors of unilateral IMC using CBCT. The results of the present study indicated that the total root length of the MLI was smaller on the impacted side. This was in agreement with studies by Kim et al. [4] and Priya et al. [11]. Among the proposed mechanisms, guidance theory, which posits that the root of the MLI significantly influences the normal eruption of the MC, has garnered acceptance as the most credible explanation. This theory asserts that any impairment in the function of the root of the MLI leads to a deviation in the eruption of the MC, thereby highlighting the critical role that the root of the MLI plays in the eruption process of the MC [12]. Previous studies have reported resorption of the root of the MLI due to prolonged contact and pressure from the IMC, which could have led to root shortening in MLI; this resorption is usually oblique and not horizontal [13,14]. This was confirmed in our study by the presence of decreased angulation between the long axis of the MLI and IMC, indicating the closeness of IMC to the roots of MLIs.

Furthermore, the MD dimension of the MLI was not significantly different on either side. This is in agreement with the study by Brin et al. [5]. Although previous studies have postulated that MLI is required to guide the eruption of MC, the presence of small or peg-shaped MLIs has been associated with IMC [3]. However, this was not observed in our study, which could have been due to the fact that in our population, IMC could have been related to other factors such as crowding, abnormal eruption paths, or lack of space in the dental arch, rather than changes in the dimensions of the adjacent MLI.

Our study reported the presence of mesially inclined MLI roots on the impacted side, depicted by the presence of increased angulation of the long axis of the MLI to the MSP. This finding is in agreement with that of Kanavakis et al. [15]. The findings from the current study may bolster either of the two dominant theoretical frameworks. In favor of the guidance theory, one could postulate that a mesially angulated root would be inadequate for facilitating the eruption of the neighboring MC. Conversely, this identical morphological anomaly may also be interpreted as a developmental irregularity on a genetic basis.

Our study also reported that MLIs were more labially inclined on the impacted side than on the non-impacted side. This could have been due to the placement of the crown of the IMC, labial to the root of the MLIs. The IMC might have exerted pressure on the lateral incisor root as it attempted to erupt, leading to a compensatory labial inclination. Altered pressure from soft tissues, such as the lips or tongue, may exacerbate the labial inclination of the lateral incisor in the absence of proper MC support. This finding is supported by Hu et al. [16].

Significant gender differences were noticed in the present study for all variables except the MD dimension of the MLI. To eliminate bias due to sex, an equal number of males and females were included in our study. It was noted that females exhibited smaller MLI dimensions (root length and LL dimension) on the impacted side. This is in agreement with the results of previous studies [9,17]. Hormonal differences between males and females during growth and development may play a role in influencing tooth size and development, with females being more prone to slight reductions in dental dimensions. Females often experience slightly earlier tooth eruption than males, which might limit the time available for complete root development. This could result in shorter root lengths, particularly in situations involving dental anomalies, such as IMC.

The results of the present study indicated that sex, angulation of MLI with respect to MSP and IMC, MD dimensions of MLI, and root length were significant indicators of unilateral IMC. Considering that the distal segment of the MLI root serves as a natural containment barrier for the initial mesial and palatal displacement of the MC, thereby facilitating its eruption, the presence of mesially displaced roots of MLIs adjacent to displaced MCs may represent a critical clinical indicator that the canine has forfeited its association with eruption guidance [18]. Similarly, small or peg-shaped MLIs were found to be associated with IMC [10]. According to guidance theory, the findings can substantiate that reduced MLI dimensions lead to a compromised eruption of the MC. It may be deduced that individuals possessing smaller crowns and roots of MLI are predisposed to aberrant eruption patterns of the MC.

Limitations

The principal constraint of the current study resides in its retrospective framework, which inhibits our ability to ascertain a genuine causal relationship. Moreover, the familial history of IMC was not evaluated in our study. It would have been advantageous to use a larger sample size. Nevertheless, the acquisition of additional CBCT images was impeded by the constraints inherent in the retrospective study design. Furthermore, the dimensions and angulations of the canine to the OP and MSP were not evaluated in the present study. Further studies should be conducted in the future.

## Conclusions

The results of the present study indicated significant sex differences in all variables except the MD dimension of the MLI. The dimensions of MLI were smaller in females than in males. Unilaterally, IMC has been significantly associated with shorter root length, LL dimension, mesially displaced roots, and closeness to MLIs. Sex, angulation of the MLI with respect to the MSP and IMC, MD dimensions of the MLI, and root length were significant indicators of unilateral IMC.
